# Protection of dogs against canine heartworm infection 28 days after four monthly treatments with Advantage Multi® for Dogs

**DOI:** 10.1186/s13071-016-1293-z

**Published:** 2016-01-08

**Authors:** Dwight D. Bowman, Alyssa R. Grazette, Chris Basel, Yingying Wang, Joseph A. Hostetler

**Affiliations:** Liberty Research, Inc., Waverly, NY USA; Bayer HealthCare LLC, Shawnee Mission, Kansas, USA

**Keywords:** Steady state, *Dirofilaria immitis*, Moxidectin, Dogs, Prevention, Pharmacokinetics, Resistance

## Abstract

**Background:**

Monthly heartworm preventives are designed to protect dogs by killing heartworms acquired the month prior to their administration, and after treatment with most products, the drug levels rapidly dissipate to very low levels. Work with Advantage Multi® for Dogs (imidacloprid + moxidectin) topical solution showed protection against hookworm infection throughout the month after administration of several monthly doses suggesting that similar protection might occur with heartworms. This study assessed the amount of protection afforded to dogs by the administration of four monthly doses of Advantage Multi for Dogs prior to infection with third-stage heartworm larvae (*Dirofilaria immitis*) 28 days after the last (fourth) treatment.

**Methods:**

There were 16 purpose-bred mongrel dogs in the study that were divided into two groups, 8 control and 8 treated dogs. Dogs were housed in a manner preventing contact between animals and groups, and personal protective gear worn by staff minimised the chance spread of the topically applied product between runs. The dogs in the treated group received monthly applications of Advantage Multi for Dogs as per label instructions on Study Days 0, 28, 56, and 84. On Study Day 112, all 16 dogs received 50 third-stage larvae of *D. immitis* (“Missouri” isolate) *via* subcutaneous inoculation in the inguinal region. The study was terminated on Day 264, and the number of heartworms per dog was determined at necropsy.

**Results:**

Moxidectin levels after 4 treatments 28 days apart were near steady state on Study Day 112 when the dogs were inoculated with *D. immitis* third-stage larvae. At necropsy, 152 days after infection, all the control dogs had adult worms in their pulmonary arteries (geometric mean = 33.9; range 25–41), and none of the dogs treated four times prior to infection, with the last treatment 30 days prior to infection, harbored worms at necropsy.

**Conclusions:**

The efficacy of prevention was 100 % when the dogs were infected 28 days after the last monthly treatment. When dogs receive consecutive doses of Advantage Multi for Dogs as prescribed, heartworm infections will be prevented throughout the monthly dosing interval after administration of several monthly doses.

## Background

Prevention of heartworm disease by monthly application of macrocyclic lactones (e.g., ivermectin, milbemycin, moxidectin) is based on their ability to kill young developing stages (third-stage and fourth-stage larvae) that have been acquired and begun their development during the month prior to treatment. Treatment early in the infection is required because older fourth-stage larvae and young and older adult worms are less susceptible to the doses that are effective against the stages that have been in the dog less than a month [1]. Pharmacokinetic studies have shown that following the oral administration of ivermectin and other products that there is a rapid clearance of these products from the serum [2, 3]. As a consequence, development of larval stages following any new infection during the month after administration is generally considered to progress normally until the next monthly treatment kills any worms newly acquired in the past month.

Advantage Multi® for Dogs (imidacloprid and moxidectin) is formulated for topical administration to provide at least 10 mg/kg imidacloprid and 2.5 mg/kg moxidectin. The moxidectin, which is the portion of the product with activity against the internal helminth parasites is approved to prevent heartworm disease by killing stages of *D. immitis* that are less than thirty days of age in the dog. This product is also labelled as efficacious in the treatment of fourth-stage larvae and adults of *Ancylostoma caninum*, *Uncinaria stenocephala*, and *Toxocara canis*, and the adults of *Toxascaris leonina* and *Trichuris vulpis*.

Additional work since product approval has shown that Advantage Multi® for Dogs and Advantage Multi® for Cats will protect pet dogs and cats against incoming hookworms for a month after their last treatment. When dogs were inoculated with 300 infective larvae the hookworm *Uncinaria stenocephala* 18 days after a single treatment with Advantage Multi® for Dogs, there were no hookworms recovered from the treated dogs 21 days after the larvae were administered, while the non-treated control dogs harbored both immature adult 4.6 (SD ± 3.9) and adult hookworms 8.1 (SD ±4.3) [4] Also, the month-long protection against incoming parasites provided by Advantage Multi can be enhanced if the pet first receives several consecutive monthly administrations. In work with canine hookworms, dogs were treated with Advantage Multi® for Dogs on days 0, 30, 60, and 90 and were then given larvae of hookworms 22 days (*U. stenocephala*) and 23 days (*Ancylostoma caninum*) after the last treatment. Examination of the intestinal tracts of the treated dogs 42 days (*U. stenocephala*) and 41 days (*A. caninum*) after larval inoculation (63 days after the last treatment) revealed no *U. stenocephala* and only one adult *A. caninum* in the six treated dogs while the non-treated control dogs examined on the same day harbored a mean of 517.8 *U. stenocephala* and 41.3 *A. caninum* adults [5]. Using the feline hookworm *Ancylostoma tubaeforme* in a similar study design, when cats were infected 20 days after the last of five monthly treatments with Advantage Multi® for Cats, there were no hookworms recovered from the treated cats, while there was a mean of 62.5 adult worms recovered from the non-treated cats [5].

Protection following the regular monthly administration of Advantage Multi has now also been reported relative to feline infections with *D. immitis* [6]. Four monthly treatments with Advantage Multi® for Cats protected cats from subsequent regular weekly infection with *D. immitis* for 28 days. These treatments prevented both the formation of a detectable antibody response and development of pulmonary lesions by either immature stages of *D. immitis* or young adult heartworms. The work here reports on the ability of Advantage Multi® for Dogs to protect against inoculated heartworm larvae that were administered 28 days after the last of four consecutive monthly doses.

## Methods

### Animals

Sixteen purpose-bred Mongrel dogs (8 males and 8 females) purchased from a USDA approved commercial Class A supplier were used in this study. The dogs were acclimated to the study environment for eight days prior to the initial treatment. Physical examinations were performed by a veterinarian on Study Day −6. General Health Observations were conducted once daily throughout the study. On Study Day −2, the 16 dogs were randomly assigned to one of two treatment groups; the animals were separated by sex, ranked by ascending body weight, and alternately assigned to treatment groups 1 or 2. All animals were individually housed in a manner that prevented contact between the animals and separated by group as a precaution against spreading of the topically applied product between animals. Temperature ranged from 11.7 to 27.6 °C, and overhead fluorescent lights on an automated timer provided a 12-h light/dark cycle throughout the study. Dogs were fed Laboratory Canine Diet (LabDiet®, St. Louis, MO) *ad libitum*, and fresh water was available to all animals throughout the study. Personnel that performed general health observations as well as those conducting the necropsy, dissection and heartworm counts were masked to treatment throughout the study. All procedures in this study were reviewed and approved by the facilities Institutional Animal Care and Use Committee and housed according to USDA Animal Welfare Regulations, Animal Welfare Act (9 CFR 1–3).

### Treatment

The treated group of dogs received Advantage Multi® for Dogs, per label instructions, assuring the minimum recommended dose of 2.5 mg/kg moxidectin and 10 mg/kg imidacloprid at days 0, 28, 56 and 84.

### Inoculation with heartworms

On Study Day 112, 28 days after the last Advantage Mutli for Dogs administration, all dogs were inoculated with 50 infective, third-stage larvae (L3) of *D. immitis* (Missouri Isolate) harvested from infected mosquitoes; this same isolate was used in the steady state trial performed with heartworms in cats [6]. Infective L3 larvae were harvested from *Aedes aegypti* (Liverpool strain) into Hanks’ Balanced Salt Solution 15 days after membrane feeding on heparinised blood containing approximately 3,500 microfilariae/ml. Fifty *D. immitis* L3 larvae were counted into Petri dishes for each individual animal. Excess fluid was removed from the dish to ensure that less than 1 mL of fluid remained in each dish. The fluid containing the larvae was then drawn into a 1 mL syringe. The contents of the syringe were injected into the animal subcutaneously in the inguinal region. The syringe was rinsed several times with Hank’s Balanced Saline Solution and reinjected to ensure all larvae were removed from the syringe. The syringe was then rinsed into a Petri dish and examined for remaining larvae. Any remaining larvae were injected in the animal.

### Determination of Moxidectin blood levels

For serum moxidectin level determination, blood samples were collected in serum separator tubes on Study Days −2, 10, 27, 39, 55, 66, 83, 91, 98, 105, 112, 119, 140, 168, 181, 210, 240 and 264. The samples were then centrifuged and the serum was harvested and stored at −80 °C. Once all samples had been collected and frozen, serum samples were shipped on dry ice to a laboratory (Bayer CropScience AG, Monheim, Germany) for determination of moxidectin levels using a validated method. Samples were first deproteinised by mixing 200 μL of serum with 800 μL of a precipitation solution and then filtered. The precipitation solution was prepared by mixing 100 mL of a solution of 0.39 g ammonium acetate in 1 L water with 1 mL of formic acid and 600 mL of acetonitrile. The quantitative determination of moxidectin in serum was performed by high performance liquid chromatography-tandem with tandem mass spectrometric detection using an AB Sciex API 4000 mass spectrometer (Bayer CropScience AG Method 01086/M001). The lower limit of quantitation was 1 μg/L.

### Heartworm antigen analysis

For heartworm antigen testing, blood was collected in tubes containing EDTA on Study Days 112, 181, 210, 240 and 264. These samples were centrifuged and the harvested plasma was stored at −80 °C. Following study completion, samples were sent to Auburn University. The samples were analysed using a DiroCHEK enzyme-linked immunosorbent assay (ELISA) for the detection of adult *D. immitis* antigen in the serum. To obtain quantitative results from this analysis, the plates were read with a Bio-Rad iMark Microplate Reader. A standard deviation of 0.005 was added to the optical density (OD) readout for the negative control and this number was used as a cutoff. Any OD readout above the cutoff was interpreted as a positive result and anything below was interpreted as a negative result.

### Post-mortem examination

On Study Day 251, all animals were randomised to necropsy order, and then on Study Day 264, all dogs were humanely euthanised, necropsied and lungs and heart were collected. The heart and lungs were then dissected and any heartworms found were collected and counted (by gender and total).

### Statistics for efficacy determination

Statistics were performed using SAS® 9.3 (SAS Institute Inc., Cary, NC, USA). The total heartworm counts from necropsy (sum of male and female worms) from two treatment groups were analysed by Wilcoxon’s Rank Sum test using a two-sided exact test based on the Wilcoxon rank-sum statistic testing for treatment group effects. An alpha of 0.05 was used to conclude a statistical significant difference.

### Pharmacokinetic evaluation

The pharmacokinetic evaluation consisted of graphical evaluation of steady state attainment and the calculation of apparent terminal half-lives. Phoenix WinNonlin version 6.3.0, developed by Pharsight Corporation, was used for graphs of individual results and half-life determination, and Microsoft Excel 2010 was used for the graphical evaluation of steady state attainment. Phoenix has been validated by Pharsight and at the site of use. Time points used for half-life determination were manually chosen.

## Results

### Efficacy of treatment

All eight (8) Advantage Multi®-treated dogs had no heartworms recovered at necropsy. The eight (8) non-treated dogs had a geometric mean heartworm count of 33.9 (ranging from 25 to 41) (Table 1). A statistical significant difference was present between the total heartworm counts in both groups (*P* = 0.00016).

**Table 1:** Worm recoveries from dogs treated with 4 monthly doses of Advantage Multi for Dogs with the last treatment being 28 days before being inoculated with L3 *Dirofilaria immitis* larvae from mosquitoes. Shown in the table are the dogs’ IDs and gender, ELISA OD value on Day 264 (152 days after inoculation with larvae), and the number of male, female, and total worms recovered from each dog

			Number of Worms Recovered		
Dog ID	Gender	ELISA OD Day 264	Males	Females	Total
DOGS TREATED WITH ADVANTAGE MULTI® FOR DOGS					
501	Male	0.055	0	0	0
701	Male	0.052	0	0	0
801	Male	0.054	0	0	0
601	Male	0.057	0	0	0
505	Female	0.060	0	0	0
705	Female	0.056	0	0	0
703	Female	0.054	0	0	0
704	Female	0.056	0	0	0
Geometric mean:			0.00	0.00	0.00
NONTREATED CONTROL DOGS					
502	Male	0.063	12	22	34
702	Male	0.055	7	18	25
306	Male	0.080*	13	21	34
401	Male	0.055	14	17	31
504	Female	0.093*	13	24	37
506	Female	0.090*	20	21	41
503	Female	0.079*	13	23	36
602	Female	0.090*	21	15	36
Geometric mean:			13.47	19.90	33.94

*The asterisk next to the OD (Optical Density) indicates these dogs were considered antigen positive because the OD reading was >0.005 above the negative control reading of 0.067

### Heartworm antigen

All dogs tested negative for heartworm antigen on Study Days 112, 181, 210, 240. All eight treated dogs remained sero-negative for heartworm antigen throughout the study. On Study Day 264, 5 of the 8 dogs in control group were antigen positive (Table 1).

### Pharmacokinetic analysis

**Fig. 1:**
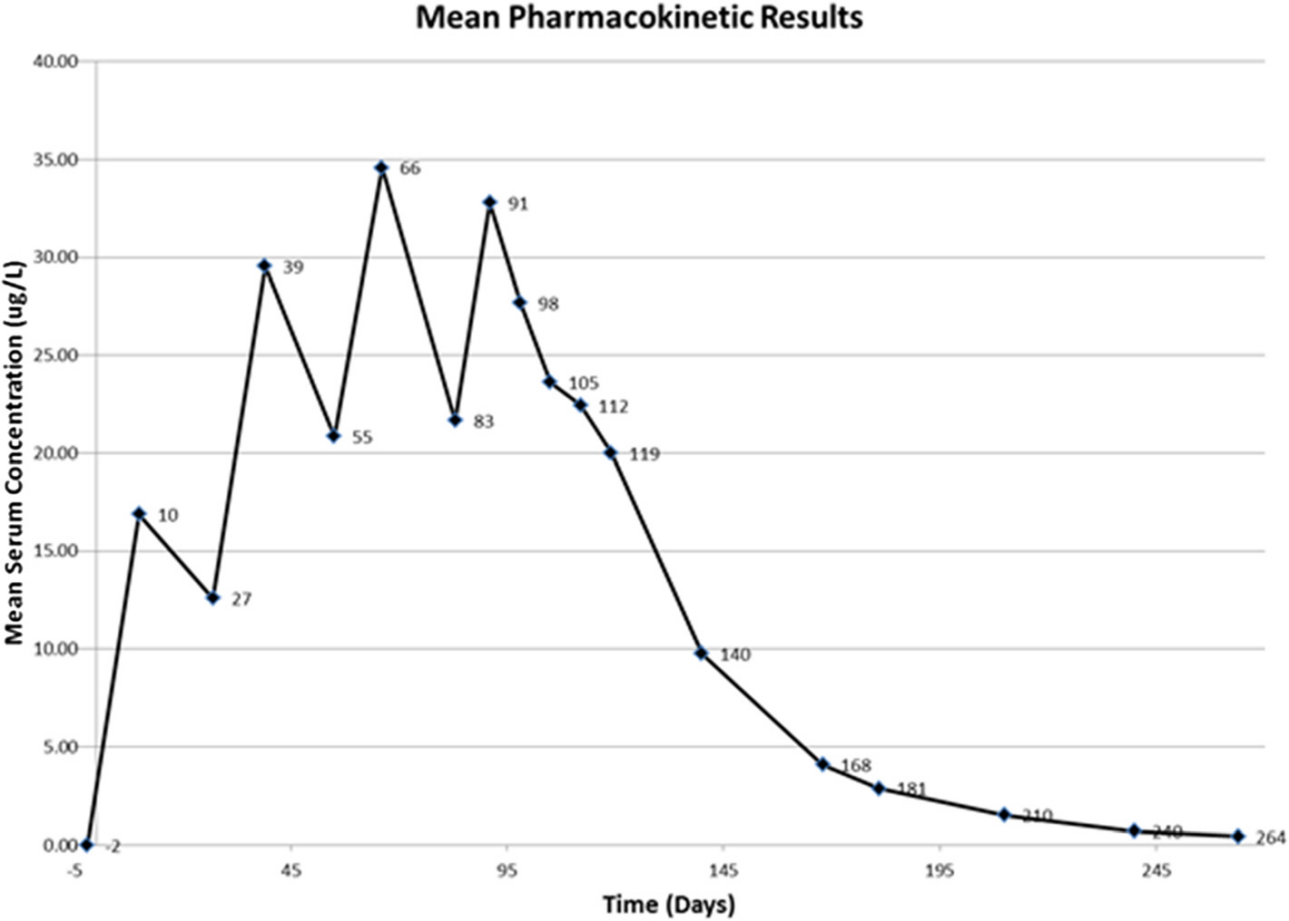
Levels of moxidectin in serum of dogs collected for analysis: 1. One or two days before each treatment (days −2, 27, 55, 83); 2. Ten or eleven days following treatments one through three (days 10, 39, 66); and 3. After the last treatment (days 91, 98, 105, 112, 119, 140, 168, 181, 210, 240, and 264). Subjects were infected with heartworms on day 112

**Fig. 2:**
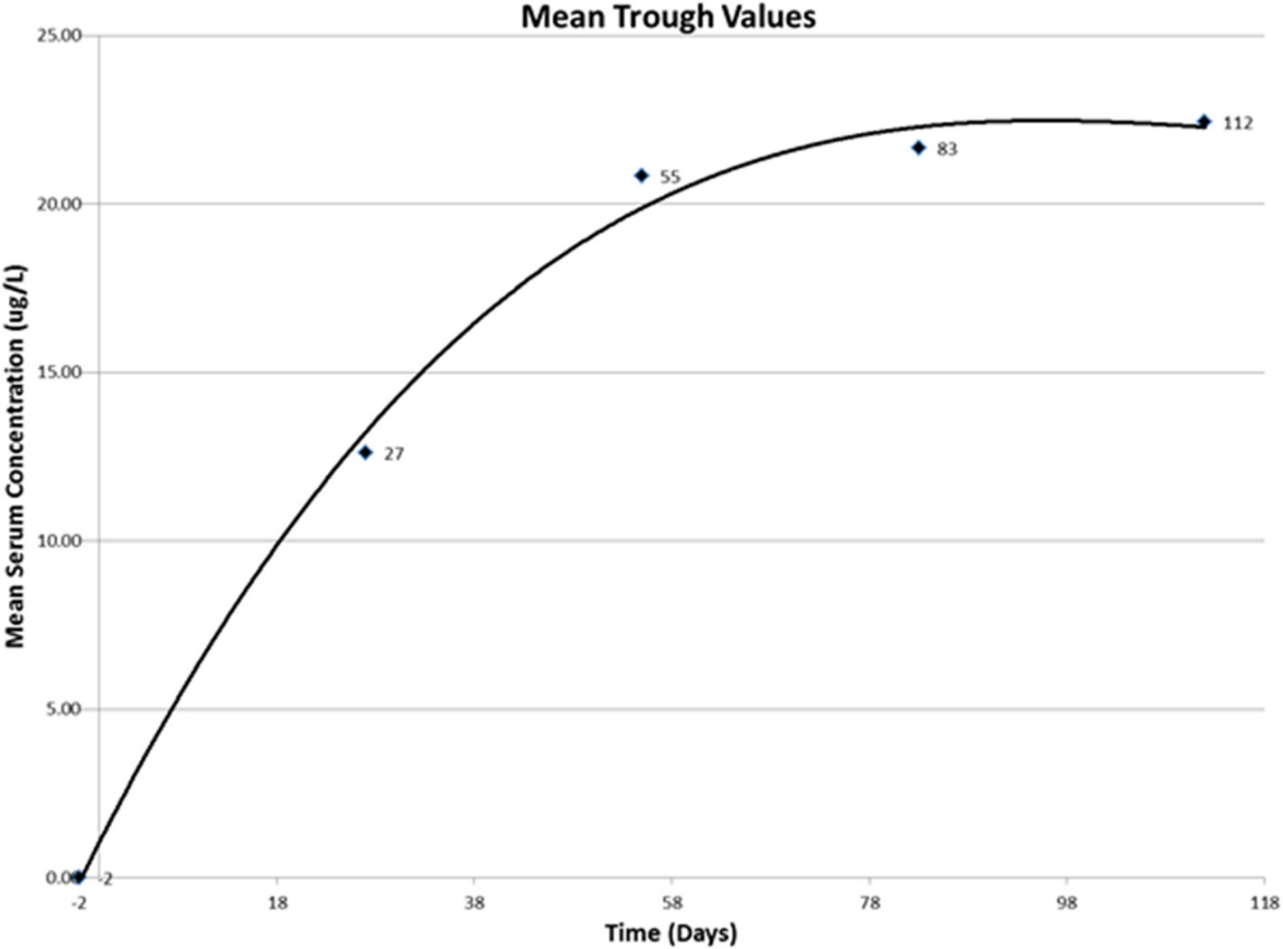
Mean trough levels of moxidectin (μg/L) prior to infection with *Dirofilaria immitis* on Day 112. Dogs were treated with moxidectin on Study Days 0, 28, 56, and 84 and inoculated with *third-stage larvae* of *Dirofilaria immitis* on Study Day 112

A mean graph of the pharmacokinetic data shows a characteristic “saw tooth” multiple dose profile (Fig. 1), with increasing concentrations with successive administrations, indicating accumulation, and eventually attainment of steady state. The moxidectin level is maintained above the C_max_ of the first administration for at least an additional 28 days after the last administration as demonstrated by the serum levels at day 112, i.e., the day on which the dogs were inoculated with *D. immitis* third-stage larvae. Examination of the mean trough values (pre-administration concentrations) more clearly shows moxidectin accumulation with attainment of steady state (Fig. 2). The mean terminal phase half-life was determined to be 28.4 days. Based on this half-life, steady state (nearly 94 %) should be reached in approximately 4 × 28.4 days, or ~114 days. This is consistent with the results observed in the graph of trough values (Fig. 2).

## Discussion

Treating dogs with moxidectin four times at intervals of 28 days provided protection against a challenge with 50 third-stage larvae of *D. immitis* 28 days after the administration of the last of the four treatments. It would appear that steady state accumulation of moxidectin provides levels that are capable of preventing the development of worms for a month after the last of the four treatments. This had previously been observed to occur in cats that were inoculated with 25 third-stage larvae of *D. immitis* 7, 14, 21, and 28 days after the last of four monthly treatments [6]. Therefore, unlike the administration of oral ivermectin at preventive heartworm doses where the drug level is markedly reduced within hours of administration [2, 7], with the topical administration of Advantage Multi for dogs, steady state is reached with the topically applied moxidectin with accumulation of moxidectin that provides at least a month of protection after the last administration to dogs that have received four or more monthly applications. The authors also presume based on the observed pharmacokinetic profile that the same protection would occur after one monthly dose due to the high serum concentration seen in the dogs in this trial.

Besides comparison with the first low-dose macrocyclic lactone used for heartworm prevention, ivermectin in HEARTGARD®-30, topically applied Advantage Multi® for Dogs appears unique when compared to the other heartworm preventives in its ability to accumulate at steady state, achieve, and maintain effective drug levels in the body even after 28 days since the last administration. The daily oral administration of the mixture of milbemycin A_3_A_4_ 5-oxime (Interceptor® Flavor Tabs® for Dogs and Cats) when given to dogs for a month and a half falls rapidly to near zero within two weeks after the last treatment [3], indicating that this formulation does not accumulate and reach effective levels at steady state as does topically applied moxidectin. The addition of spinosad to milbemycin oxime within Trifexis®, does increase the plasma levels of milbemycin oxime in treated dogs, but even after three monthly treatments, the levels are reduced by three orders of magnitude within 25 days [8]. Also, it appears that accumulation is not sufficient to create effective levels of selamectin when applied as Revolution. Topically and orally dosed selamectin levels fall much slower in dogs following a single application than after intravenous administration; however, the levels fall much more rapidly than for topically applied moxidectin [9]. With the sustained release formulation of moxidectin, Proheart® 6, as stated in NADA 141–189 ProHeart® 6 (moxidectin) Sustained Release Injectable for Dogs: “Following injection with the 1X dose of ProHeart 6, peak moxidectin blood levels were observed approximately 7–14 days after treatment. By Day 112 after the first treatment, moxidectin serum levels were below the limit of quantification (0.5 ppb) in 10 of the 12 treated dogs. Measurements taken prior to the fifth and sixth injections showed consistently low moxidectin serum levels. Accordingly, little or no drug accumulation occurred with repeated administrations.” [10]. Again, it appears that topical moxidectin is unique in its bioaccumulation of preventive levels of anti-filarial activity by the simple monthly topical application of the product.

Recently, it has become apparent that there are isolates of heartworms that are not killed by the single doses of preventive products as they once were ([11–17], McCall *et al.*, 2013, unpublished “late breaking” poster, 14th Triennial Symposium of the American Heartworm Society, Sept 8–10, New Orleans, LA, USA). However, some of the products have been found to have increased efficacy against inoculated larvae when administered monthly for several months after infection, e.g., Trifexis® and Sentinel® Spectrum® Chewables® (NADA 141–333) [16, 18]. It is unclear why these repeated treatments are efficacious, because the results of the original approvals for HeartGard-30 (NADA 138–412) and Interceptor® (NADA 140–915) showed that heartworms became more refractory to macrocyclic lactone effects as they aged [1, 19]. It now appears that perhaps the repeated insult to developing worms from successive treatments may prevent their development as they are repeatedly treated after being introduced into dogs. The susceptibility of these developing forms to repeated dosages of macrocyclic lactone suggests that the constant high levels of moxidectin in the blood of dogs on regular monthly Advantage Multi® for Dogs may increase the chance of it killing these incoming worms that are no longer killed by the single dose of low-dose macrocyclic lactone. If one examines the curve of the mean pharmacokinetic results (Fig. 2), it can be seen that the treatment levels with Advantage Multi® for Dogs are actually higher after 2 or 3 applications than after a single application, and topical moxidectin has had excellent activity against less susceptible heartworm isolates with only a single application (McCall *et al.*, 2013, unpublished “late breaking” poster, 14th Triennial Symposium of the American Heartworm Society, Sept 8–10, New Orleans, LA, USA) ([11, 12], McCall *et al.*, 2013, unpublished “late breaking” poster, 14th Triennial Symposium of the American Heartworm Society, Sept 8–10, New Orleans, LA, USA). Thus, as topically applied Advantage Multi® for Dogs reaches steady state after several applications, it may be that when the high level of moxidectin is available it will kill all new infections of susceptible isolates. This may even be efficacious against the less susceptible isolates that require several consecutive monthly treatments for prevention to occur. This is something that might be worthy of testing experimentally. At this point in time, it can be speculated that the continued year-round application of monthly topical moxidectin in the form of Advantage Multi® for Dogs may be a means of protecting dogs against these new resistant isolates of *D. immitis*.

## Conclusions

The administration of several monthly applications of moxidectin in topically applied Advantage Muti® for Dogs provides levels of moxidectin that are protective against incoming third-stage larvae of *D. immitis* for at least a month after the last dose is administered. The pharmacokinetic profile suggests that this may even be the case after only a single dose of topical moxidectin as formulated in Advantage Multi® for Dogs.
